# First-in-human application of dynamic fluoroscopic analysis to quantify intersegmental motion in mandibular free flap reconstruction

**DOI:** 10.1038/s41598-025-22244-3

**Published:** 2025-10-20

**Authors:** Henri Kreiker, Philippe Moewis, Claudius Steffen, Philipp Ruf, Georg N. Duda, Sara Checa, Max Heiland, Jakob Fenske, Carsten Rendenbach

**Affiliations:** 1 Department of Oral and Maxillofacial Surgery, Charité – Universitätsmedizin Berlin, Corporate Member of Freie Universität Berlin and Humboldt-Universität zu Berlin, Berlin, Germany; 2 Julius Wolff Institute, Berlin Institute of Health at Charité – Universitätsmedizin Berlin, Berlin, Germany; 3 Institute of Biomechanics, Hamburg University of Technology, Hamburg, Germany

**Keywords:** Mandibular reconstruction, Fluoroscopy, Intersegmental movement, Regeneration, Fibula free flap, Tantalum beads, Model-based roentgen stereophotogrammetry, Diseases, Health care, Medical research

## Abstract

Osseous non-union following free flap reconstruction of segmental mandibular defects can prolong patients’ dental rehabilitation. Various plating systems have been developed to optimize biomechanical fixation, but healing may be retarded. Quantifying intersegmental micromovements could help monitor healing but remains challenging. This study investigates a novel method to visualize segmental movements during healing using a fluoroscopy-based approach. To track segment movements, tantalum beads were implanted intraoperatively in the osseous flap and native mandibular segments. Additionally, single-plane fluoroscopic imaging was performed to assess bead position at maximum mouth opening and intercuspation. Bead positions were merged as three-dimensional objects. Intersegmental movements were quantified using model-based roentgen stereophotogrammetry (mbRSA). Exemplarily, preliminary images were collected from one patient. Fluoroscopic imaging with mbRSA effectively displayed movements and allowed quantification. Translation and rotation were assessed between the native mandible and the flap during maximum mouth opening and intercuspation. For the first time, our analyses demonstrate the feasibility of quantifying segment mobility during healing. This first in men study illustrates the feasibility of the method to monitor intersegmental movements in cases of maxillofacial reconstructions. Further research involving larger patient cohorts is necessary to identify relevant thresholds and differentiate from those that result in lack of healing.

## Introduction

Autologous osseous free flaps, such as fibula, deep circumflex iliac artery (DCIA) and scapula free flaps, are the gold standard for reconstructing segmental mandibular defects, enabling functional and biomimetic oral rehabilitation. Modern approaches utilizing computer aided design and manufacturing (CAD/CAM) facilitate time-efficient, predictable, and precise treatments^1–3^. However, complications such as osseous non-union between segments persist, prolonging patient recovery^4^. Non-unions often impede patients’ chewing functional rehabilitation because dental implants cannot be placed in such situations. Osseous complication rates are as high as 50%^5^.

Besides clinical factors such as radiotherapy, inadequate mechanical stimuli are thought to be mainly responsible for these non-unions. Especially, the initial healing phase is sensitive for suboptimal intersegmental gap movements^6^. Understanding the correlation between intersegmental movements and the occurrence of osseous union or pseudarthrosis formation could lead to improved biomechanical fixation systems.

Consequently, clinical and biomechanical evaluations have led to the development of novel plating systems aimed at balancing ossification-promoting intersegmental stimuli^7–11^. Imaging of micromovements between osseous segments is crucial to refining reconstructive methods. However, no current in vivo bone imaging system is available, and existing methods measuring rotational movements of long bones are too sensitive to soft tissue movement artifacts^12,13^.

Fluoroscopy, a real-time X-ray-based imaging modality widely used in interventional radiology^14^, has been employed in orthopedic research to quantify intervertebral and knee joint movements^15–17^.

Here, we adapted a single-plane fluoroscopic approach in combination with intraoperatively bone-implanted tantalum beads^18^ and model-based roentgen stereophotogrammetry (mbRSA)^19^ in an attempt to quantify intersegmental movements to monitor bone healing and consolidation in three dimensions after segmental reconstructions with fibula free flaps as partially described for joint models^17^. To the best of our knowledge, fluoroscopy and mbRSA have not yet been used to image and quantify spatial intersegmental movements in maxillofacial reconstructive surgery. In fact, methods to quantify micromovements in maxillofacial reconstructions are currently lacking. We therefore introduce this novel approach in an attempt to quantify intersegmental micromovements in mandibular one-segmental reconstructions combining fluoroscopy and mbRSA.

In this single-patient pilot study, we report details of the used methodology as well as preliminary results from the first patient, and discuss the systems’ strengths, as well as challenges to be addressed in the future.

## Methods

### Surgical procedure and baseline imaging

Ethical approval was obtained by the Charité – Universitätsmedizin Berlin ethics committee (EA1/062/21). This study was performed in accordance with the Declaration of Helsinki. Patients were considered for participation, if they were planned for reconstruction of one-segmental mandibular defects with fibula free flaps. After receiving detailed study information by the principal investigator prior to treatment, participants provided oral and written informed consent to participate in this study. Here, a 69-year-old male patient without relevant comorbidities or prior therapy undergoing primary reconstruction of a one segmental, left-sided mandibular defect with a fibula free flap following resection of oral squamous cell carcinoma participated. The patient had a prosthetically restored, full dentition to both second molars in the upper jaw and a shortened prosthetically restored dentition in the lower jaw following resection (to the first premolar in the third, and to the first molar in the fourth quadrant.

To mark the free flap and native mandibula segment in vivo positions for later analysis, a minimum of five biocompatible, unalloyed 1 mm F560 tantalum beads (X-medics A/S, Brøndby, Denmark) were implanted in the anterior mandibular segment (AM), fibula free flap (FFF) and posterior mandibular segment (PM)) in three dimensions intraoperatively as previously described for pelvic models^18^. The beads are biocompatible and do not need to be removed. Beads were randomly positioned in previously drilled 1.2 mm bone cavities across the bone segment surfaces and secured using bone wax. The free flap was fixated using 3D-printed, patient-specific (3D-PSI) titanium plates as well as corresponding cutting and drilling guides (KLS Martin SE & Co. KG, Tuttlingen, Germany). A 2.0 mm reconstruction plate and two 1.0 mm mini plates were used for the posterior and anterior flap fixation, respectively, as previously described^8^. Further surgical procedures were performed in typical manner. The reconstruction results and the bead positions were postoperatively evaluated using orthopantomography (OPTG) and cone-beam computed tomography (CBCT).

### Fluoroscopy

Fluoroscopy was conducted three weeks postoperatively with the patient in an upright sitting position. This time point was determined based on clinical criteria: patients are typically mobile and recovered enough after three weeks to undergo the described procedure, while bone healing has commonly not advanced to mineralization stage, thereby allowing for quantification of micromovements. To correct image distortion, the fluoroscopic system was calibrated using a Perspex calibration box (BAATEngineering B.V., Hengelo, The Netherlands) prior to image acquisition as described in previous studies^20,21^.

**Fig. 1:**
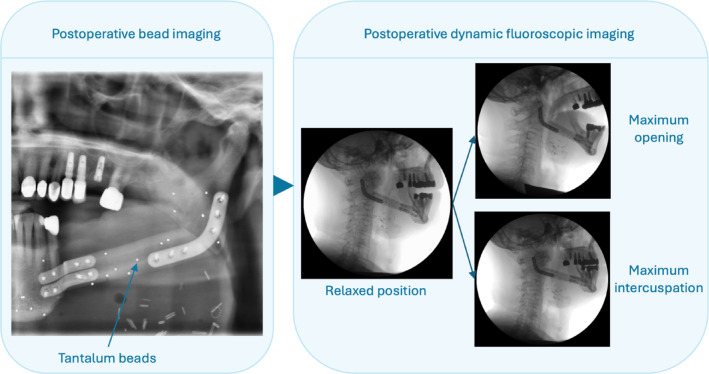
Postoperative and fluoroscopic Imaging. Postoperatively, tantalum beads were identified using routine orthopantomography. After three weeks, fluoroscopy was acquired for maximum opening and maximum intercuspation starting from a relaxed position, respectively.

Following instructions and practice, the patients performed maximum jaw movements, starting from a resting position: (a) maximum mouth opening and (b) maximum intercuspation. Fluoroscopic imaging was performed using a C-arm fluoroscope (Philips BV Pulsera, Philips Medical Systems DMC GmbH, Hamburg, Germany) with an acquisition frequency of 30 Hz. Each measurement was recorded three times (Fig. 1). During fluoroscopy, the patient received a total effective dose of 0.4 mSv (dose-area-product: 2830 mGy*cm^2^)^22^.

### Image processing and model-based roentgen stereophotogrammetry

**Fig. 2:**
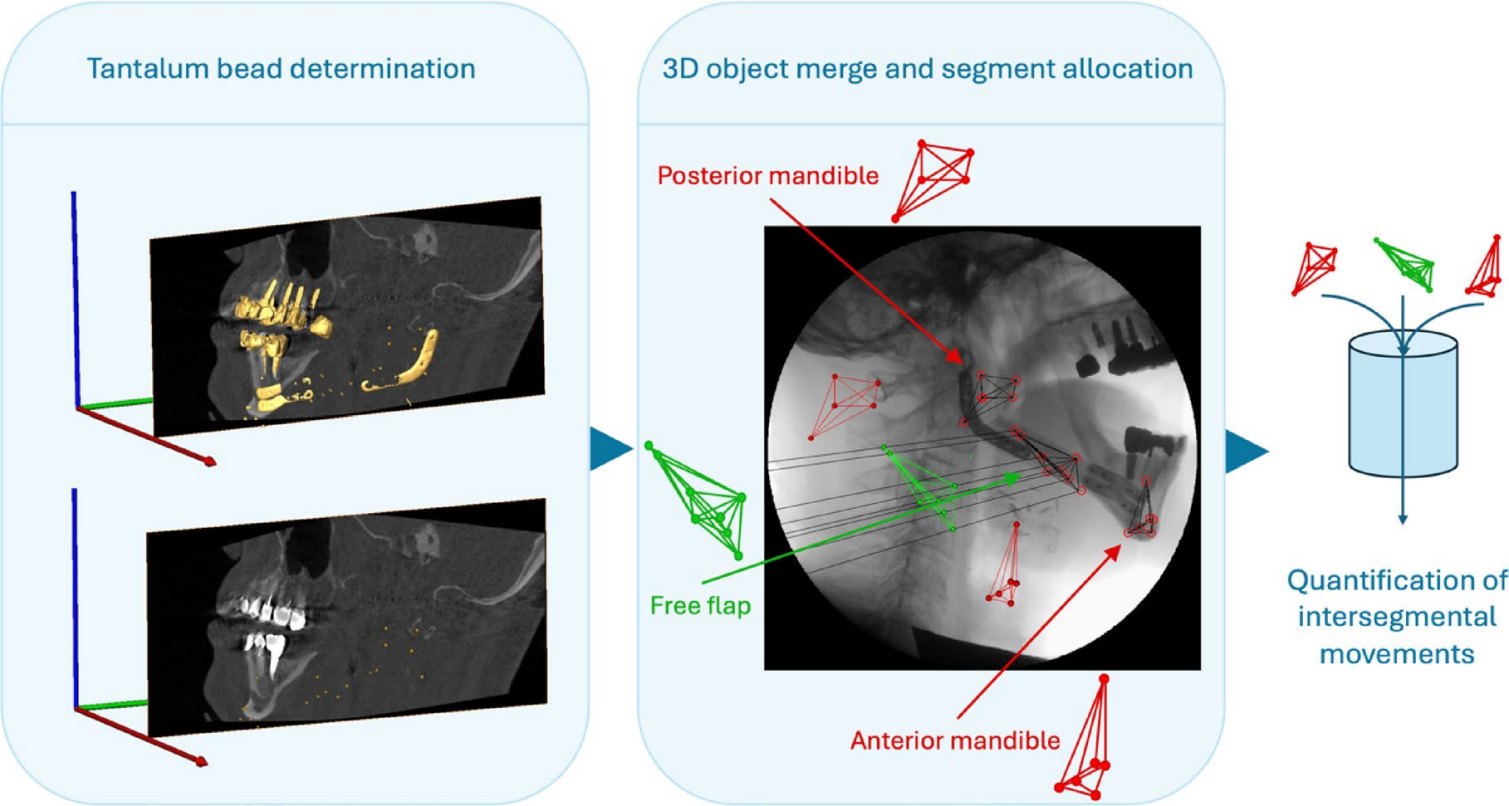
Image processing procedure. Tantalum bead positions were identified using cone-beam computed tomography. Beads were merged into 3D objects and allocated to the bone segments. Using model-based roentgen stereophotogrammetry, translational and rotational intersegmental movements were quantified.

Tantalum beads were identified in the postoperative CBCT and OPTG and assigned to the respective segments. Using the software Amira (Amira, Visage Imaging, Berlin, Germany), metallic objects were displayed in the CBCT based on thresholds and the tantalum beads were extracted. The position of each individual tantalum bead was defined and merged within the coordinate system of a separate 3D frame assigned to each of the respective segments (AM, FFF or PM). For each measurement, specific image files corresponding to the resting position and a maximum movement were extracted from the fluoroscopy. These image files and 3D frames were transferred to the commercially available software Model-based RSA (Medis specials B.V., Netherlands) for all subsequent analyses^21,22^. The 3D frames assigned to the respective segments (AM, FFF or PM) could subsequently be superimposed on the scene file in x (anterior–posterior)-, y (superior-inferior)- and z (medio-lateral)-axis. Considering that the z-axis represents the sensitive out of image-plane direction (error in relative position and orientation up to 0.8 mm and 0.6°^23^), translational changes in this direction were approximated to 0 mm. Rotational changes around the x- and y-axes were also neglected due to the aforementioned out of image-plane error. Thereby, these bead-derived 3D-objects acted as segment surrogates to facilitate quantification. Finally, the relative spatial position change between the 3D frames was measured for translational and rotational movements in the respective axes, with negative values indicating counterclockwise rotations or diverging translations (Fig. 2).

Data management and descriptive statistical calculations were performed in Microsoft Excel (Microsoft Corp., Redmond, WA, USA). Visualizations were created using the Python Programming Language (Google Colab).

## Results

**Fig. 3:**
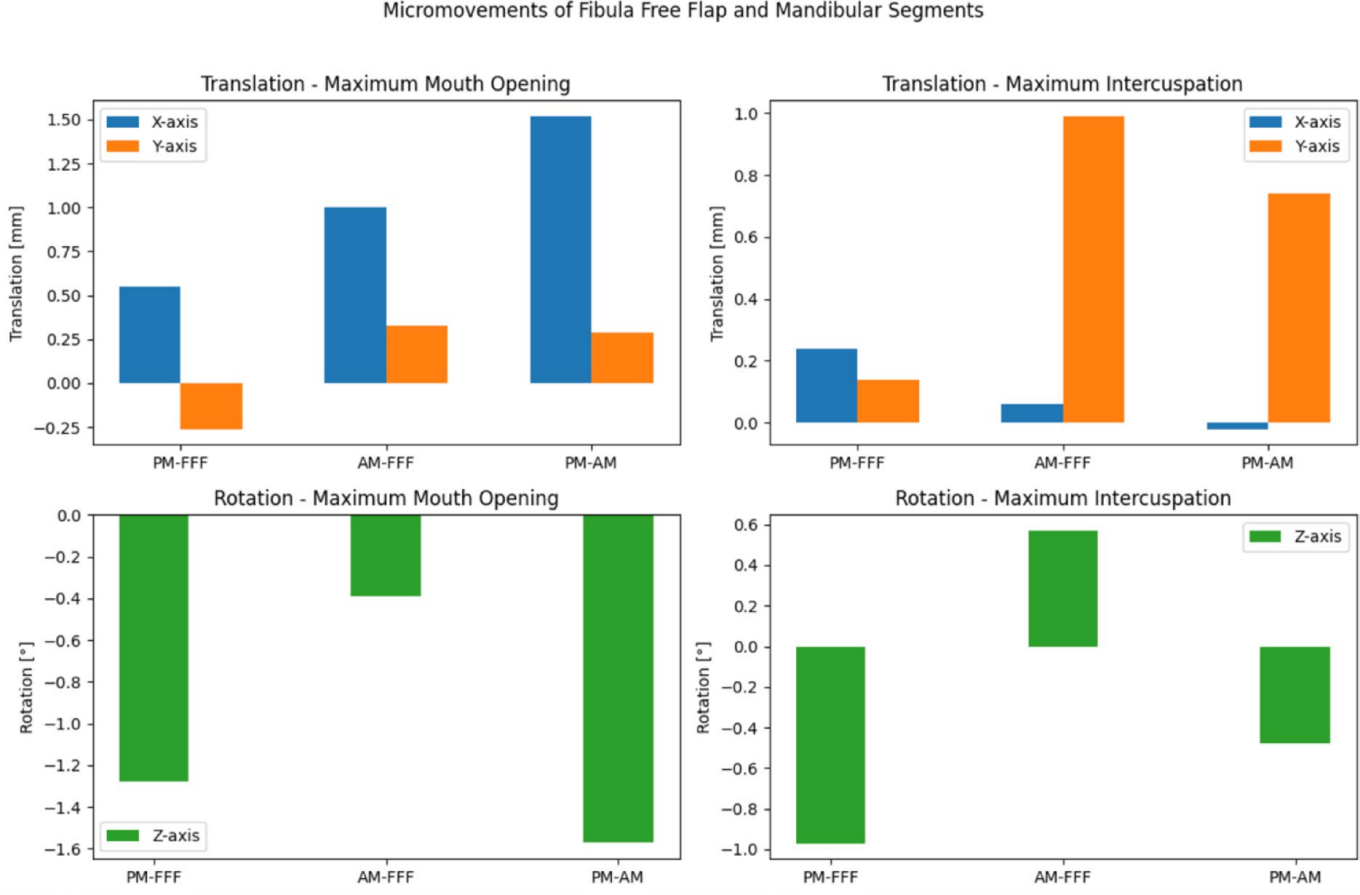
Micromovements during translation and rotation. (PM = posterior mandibular segment, FFF = fibula free flap, AM = anterior mandibular segment).

Translational and rotational movement between all segments for each maximum mouth opening and intercuspation were quantified. Data from the third measurement were suspected to contain many outliers compared to the first two measurements due to varying distribution. Calculation of absolute and relative deviation of measurement three from means of the other measurements revealed high divergence. Accordingly, results of measurement three were only included if relative deviation to means of measurement one and two was < 50% and otherwise deemed outliers and excluded (Table 1). Fluoroscopic and model-based roentgen stereophotogrammetry analysis quantified translational and rotational micromovements between the posterior mandible (PM), anterior mandible (AM), and fibula free flap (FFF) during maximum mouth opening and maximum intercuspation. During mouth opening, the FFF exhibited approximating movement towards both the AM (1.00 mm, x-axis) and the PM (0.55 mm, x-axis). The PM and AM also approximated each other (1.52 mm, x-axis). In rotation, the FFF showed an anterior-inferior tilt (Rz: − 1.28°) to the PM and a less pronounced anterior-inferior tilt to the AM (Rz: − 0.39°). During intercuspation, superior-inferior displacement reversed, with the FFF moving superiorly relative to the PM (+ 0.14 mm vs. − 0.26 mm in mouth opening, x-axis) but was consistent in direction between AM and FFF, and PM and AM with both approximating each other. The PM and AM showed slight divergence (− 0.02 mm, x-axis), again reversing the observed movements during maximum opening (+ 1.52 mm, x-axis). Rotational movements included anterior-inferior tilts of the FFF towards both the PM and the AM (Rz: − 0.97° for PM-FFF; Rz: − 0.48° for AM-FFF) (Fig. 3).

**Table 1:** Translational and rotational movements between the respective segments during maximum mouth opening and intercuspation.

	Translation x-axis [mm ± SD]	Translation y-axis [mm ± SD]	Rotation z-axis [° ± SD]
Maximum mouth opening			
PM—FFF	0.55 ± 0.67	− 0.26 ± 0.10	− 1.28 ± 0.32
AM—FFF	1.00 ± 0.50*	0.33 ± 0.75	− 0.39 ± 0.07
PM—AM	1.52 ± 0.03	0.29 ± 0.77	− 1.57 ± 0.28
Maximum intercuspation			
PM—FFF	0.24 ± 0.05	0.14 ± 0.26	− 0.97 ± 0.22*
AM—FFF	0.06 ± 0.20	0.99 ± 0.99	0.57 ± 0.51
PM—AM	− 0.02 ± 0.33	0.74 ± 1.09	− 0.48 ± 0.74

Values are reported as [mm] ± SD for translation and [°] ± SD for rotation. Negative values indicate counterclockwise rotation or diverging translation. Asterisks indicate values where measurement three was included due to relative deviation < 50%. (SD = standard deviation, PM = posterior mandibular segment, FFF = fibula free flap, AM = anterior mandibular segment).

## Discussion

While monitoring and optimizing the outcome of intersegmental ossification in mandibular reconstruction has been extensively researched, valid methods for monitoring intersegmental micromovements are yet to be established. In this pilot study, we present promising initial results of a novel technique using fluoroscopy to quantify intersegmental gap dynamics following segmental reconstruction. The results of this first-in-men application show that combining fluoroscopy and mbRSA is a feasible method for real-time quantification of intersegmental micromovements in segmental mandibular reconstructions. In future trials, this method may be applied to larger cohorts to detect clinically relevant thresholds.

The biomechanics of the mandible, notably involving torsional moments and arch narrowing during mouth opening^20,24^ as well as complex bending and rotation during biting^25^, are unique. However, this system can be interrupted following segmental resection and reconstruction, especially during initial healing^6^. The presented results indicate that prior to ossification, the mandibular segments and the flap show distinct spatial movements during maximum movements. In detail, the FFF seems to translate and rotate in relation to the PM and AM on a millimeter-scale. The observed movements, mainly including the convergence and torsion-like deviation of the FFF to the AM/PM during mouth opening and an approximately reversed translational shift during biting, seem logical when imaginatively comparing them to the dynamics of the native mandible^20,24,25^. Consequently, the applied combination of fluoroscopy and mbRSA as a real-time quantification approach to measure dynamic intersegmental movements demonstrates a feasible methodological advance, which may be applied to various reconstruction designs in patients with different medical histories in future trials. Nevertheless, the different extent and standard deviation of movements can also be interpreted as indicators for a certain instability of the fixation result, without necessarily indicating a clinical relevance. In fact, the need for certain strains has been sufficiently shown to be necessary for general, and specific mandibular bone healing^6,26,27^. To draw conclusions from the observed parameters, the proposed methodology needs to be validated on a larger subset of patients to correlate values with clinical parameters, such as osseous non-unions and pseudarthroses, bite force values or other adverse healing events.

In this study, we examined micromovements on a mixed osteosynthesis plate design, i.e. a 3D-PSI reconstruction plate in the posterior and two 3D-PSI mini-plates in the anterior gap. This set-up results from in silico and clinical biomechanical analysis, that revealed this combination to beneficial for ossification and healing outcomes^7,8^. In detail, the suspected higher mechanical load on the anterior gap, resulting in higher rates of osseous non-union, could be mitigated using this strategy. In fact, the amount of translation during mouth opening was higher between the AM and FFF in comparison to PM and FFF, while no clear tendency was visible during maximum intercuspation. Rotational movements resulted in a tendency towards slightly higher amounts between PM and FFF.

To validate these findings, future efforts should be made to internally and externally validate the results by including more patients with this, as well as other reconstruction types. Additionally, inclusion of more patients would mitigate the statistical challenges this study presented, since various outliers were found in the third measurement and re-measurement was not possible due to violation of the study protocol.

Lastly, future challenges remain and need to be addressed but also pose opportunities to take objectification to the next level and monitor reconstructive results precisely from a microscopic view. In detail, four central aspects should be addressed. First, besides one-segmental unilateral reconstructions, other dynamics could be observed in two- or even three-segmental and bilateral defects^28^. This aspect could possibly also be mitigated by the second challenge: multi-planar registration. Currently, single-plane fluoroscopy limits the feasibility to quantify the third dimension without having to approximate it virtually. A set-up used by Zhang et al. to three-dimensionally measure bone movements in the knee could be applied on the mandible as well^17^. Thereby, the single-plane limitation could be addressed to further refine the multidimensional measurement of intersegmental gap movements. Moreover, real-time MRI could be included as well if plate artifacts are further reduced by using different biomaterials such as magnesium, which would also lead to reduced radiation exposure^29,30^. Although some researchers argued limited joint mobility in MRI^31^, others successfully used it to quantify movements of the temporomandibular joint^32,33^. While these methods may be complex to initiate and would need to consider the unique mandibular dynamics, they could give valuable information about the micromovements in complex reconstructions. Third, although progressing ossification may be a limiting factor, longitudinal imaging of micromovements, especially during multimodal treatment, would be valuable to display changes over time. Lastly, larger cohorts, possibly also including comparisons to other fixation methods such as solely reconstruction plates or different plates of different thickness, are needed to validate and successfully implement this method in the future.

In summary, the results of this novel method present promising, however exploratory, finding and a feasible method to quantify and explore intersegmental movements in mandibular reconstruction with great potential for future applications.

### Limitations

While this pilot study introduces preliminary results of a promising novel method, it is not without limitations. First, this proof of principle of this novel methodology is based only on one patient, leading to an exploratory and hypothesis generating result interpretation. Second, as this technique is a newly implemented method for quantifying micromotions, which have not yet been quantified by other techniques in vivo, there is an absence of validation against established ground truth measurements. Third, calibration and superimposition of the bead position in z-axis is approximated due to the missing second fluoroscopy plane. This also results in translational movement only being quantifiable in the x- and y-axis. Fourth, fluoroscopic images are distorted and although a correction method was applied, marginal distortional effects cannot be excluded. Additionally, distinct inaccuracies in spatial resolution cannot be ruled out due to temporary bead overlay during movement. Fifth, no long-term data on potential bead migration due to bone remodeling are currently available. Sixth, patients are exposed to x-rays during the measurements. Finally, this pilot study does not analyze or answer the clinical relevance of the observed movements, which needs to be further analyzed in future prospective studies.

## Conclusion

In this first-in-men pilot study of fluoroscopic imaging of intersegmental movements as a novel method to quantify biomechanical results of mandibular reconstruction, practical feasibility and preliminary results, implying multidimensional-movements of the flap to the mandibular segments, are presented. By applying the described method, the feasibility of detecting micromovements in mandibular reconstruction was demonstrated. Although technical refinements are necessary and patients are exposed to radiation, this method seems to be a promising approach to further study the biomechanics of mandibular reconstructions. In future applications, this method must be validated in larger cohorts to identify clinically relevant thresholds.

## Data Availability

The data used for the creation of this manuscript will be made available by the corresponding author upon reasonable request.
